# Circulating CTRP9 and aortic valve calcification jointly predict coronary artery calcification in coronary heart disease patients

**DOI:** 10.3389/fnut.2026.1783380

**Published:** 2026-04-29

**Authors:** Yanru Duan, Xiaoxue Jin, Yanan Ma, Wei Sun, Lei Nie, Shizhen Zhang, Rui Lu, Wei Cui, Guoqiang Gu, Yunhui Du, Demin Liu

**Affiliations:** 1 Beijing Anzhen Hospital, Capital Medical University, Beijing Institute of Heart, Lung and Blood Vessel Diseases, Beijing, China; 2 Department of Cardiology, The Second Hospital of Hebei Medical University, Shijiazhuang, China; 3 Key Laboratory of Medical Biotechnology of Hebei Province, Shijiazhuang, China

**Keywords:** aortic valve calcification, biomarker, C1q/tumor necrosis factor-related protein 9, cardiovascular disease, coronary artery calcification

## Abstract

**Background:**

Vascular calcification predicts adverse cardiovascular outcomes. CTRP9, an adiponectin paralog, has vascular protective effects. We evaluated the association of circulating CTRP9 with aortic valve calcification (AVC) and coronary artery calcification (CAC) in a cross-sectional observational study.

**Methods:**

Nine hundred coronary heart disease patients underwent echocardiography and optical coherence tomography; serum CTRP9 was measured in a subset. Logistic regression identified CAC determinants. ROC analysis assessed CTRP9 alone and combined with AVC for predicting severe CAC. AVC was modeled in ApoE−/− mice fed a Western diet for 24 weeks, with calcification markers analyzed via PCR and Western blot.

**Results:**

CTRP9 independently protected against CAC (OR = 0.156, 95% CI: 0.11–0.21, *p* < 0.05). CTRP9 predicted CAC severity (AUC = 0.74), and combining it with AVC improved prediction of severe CAC (AUC = 0.843). Kendall’s Tau-b indicated strong correlation between severe AVC and CAC (τb = 0.597, *p* < 0.001). Exogenous CTRP9 attenuated aortic valve calcification in mice.

**Conclusion:**

Lower CTRP9 levels associate with advanced AVC and greater CAC severity. Combining CTRP9 with AVC enhances risk prediction, highlighting CTRP9 as a potential biomarker and therapeutic target in vascular calcification.

## 1 Introduction

Coronary artery disease (CAD) remains one of the leading causes of morbidity and mortality worldwide, representing a substantial burden on public health systems. Among the various pathological manifestations of CAD, coronary artery calcification (CAC) has been widely recognized as a hallmark of advanced atherosclerotic plaque progression and an important predictor of adverse cardiovascular events. Extensive calcification not only compromises the image quality and diagnostic accuracy of multi-slice computed tomography (MSCT) but also increases the technical difficulty and failure rate of percutaneous coronary intervention (PCI), while elevating the risk of acute vascular complications during the procedure (1). However, validated serum biomarkers or imaging indicators for early detection of calcification progression and risk prediction are lacking, limiting timely intervention.

Investigations into the pathogenesis of coronary artery calcification (CAC) have revealed notable similarities with aortic valve calcification (AVC). Both conditions share early-stage atherosclerotic cardiovascular disease (ASCVD) risk factors (2–6), and the initial pathological changes in calcified aortic valves resemble those in coronary arteries (7), suggesting a potential mechanistic link. Prospective data from the Multi-Ethnic Study of Atherosclerosis (MESA) indicate a significant association between AVC and CAC (8), with further studies showing that most AVC patients also exhibit higher CAC scores compared to those without AVC (9, 10). However, only 25–40% of individuals with calcific aortic valve stenosis have significant coronary artery disease (11), implying distinct pathogenic drivers. Further research is needed to clarify whether AVC presence and severity can serve as predictors of CAC and its progression.

Research in vascular imaging, cell biology, and molecular biology has established that chronic inflammation is a key factor in the development of vascular calcification (12). Furthermore, calcific aortic valve disease (CAVD) is recognized as an active inflammatory atherosclerotic process (13), emphasizing the importance of inflammation in both AVC and CAC. C1q/tumor necrosis factor-related protein 9 (CTRP9), secreted by cardiac endothelial cells, modulates inflammatory pathways, reducing vascular inflammation and atherosclerosis (14–16). Nobuhiko Miyatake et al. demonstrated CTRP9’s protective role in preventing aortic calcification in kidney transplant recipients (17), while our prior research identified CTRP9 as a potential protective factor and diagnostic marker for CAC (18). Additionally, Sweeney et al. observed a negative correlation between adiponectin levels and AVC incidence and severity (19). Given the structural similarity between CTRP9 and adiponectin, we propose that CTRP9 may also play a role in the progression of both CAC and AVC, warranting further investigation.

Therefore, this study aims to further investigate the relationship between AVC and its severity, CTRP9 levels, and CAC and its severity, with the goal of providing a foundation for the diagnosis and assessment of CAC.

## 2 Materials and methods

### 2.1 Study population

**Figure 1:**
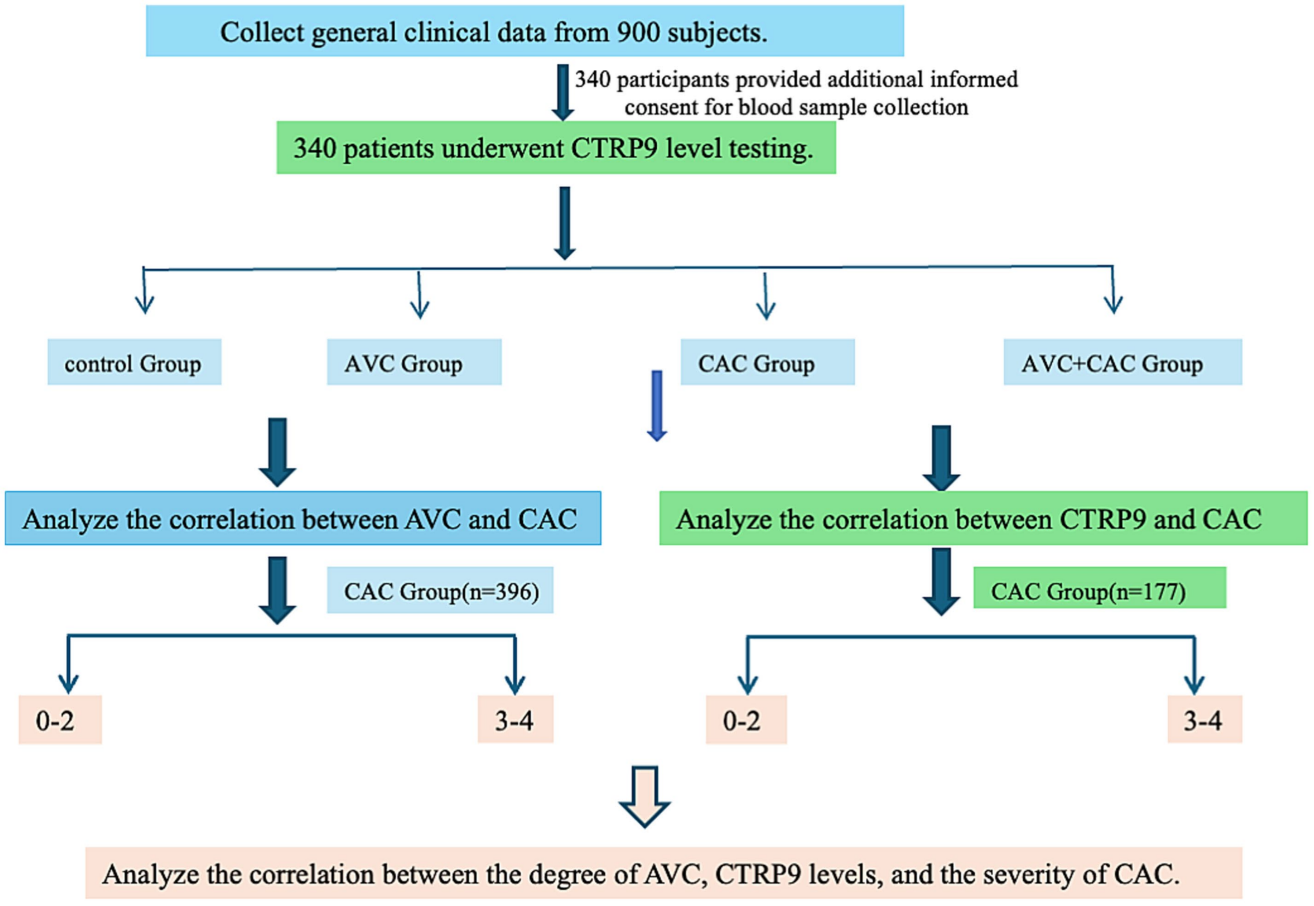
Flowchart of the clinical study design. A total of 900 subjects provided general clinical data, and 340 of them additionally consented to blood testing for CTRP9 measurement. These participants were grouped for analyses of aortic valve calcification (AVC), coronary artery calcification (CAC), and combined AVC plus CAC. CAC severity was further categorized by calcification score into lower-score and severe-score groups. The diagram shows the overall study workflow used to examine the relationships among serum CTRP9 levels, AVC status, and CAC severity in the study population.

A total of 900 eligible coronary heart disease patients who visited the Department of Cardiology at the Second Hospital of Hebei Medical University from April 2021 to July 2023 were selected according to inclusion and exclusion criteria. Among them, 340 participants provided additional informed consent for blood sample collection and long-term storage, and serum samples were successfully obtained for subsequent biomarker analyses. Therefore, all serum biomarker measurements, including CTRP9, were performed in this subgroup of 340 participants who provided serum samples. CAC patients were further divided into mild (0–2 points) and severe (3–4 points) calcification subgroups using the CVI calcification scoring system from OCT images. The study was approved by the Human Research Ethics Committee, and informed consent was obtained from all participants (Ethics approval No. 2022-C026) (Figure 1).

### 2.2 Inclusion and exclusion criteria

Inclusion criteria: coronary heart disease was diagnosed according to the 2019 ESC Guidelines for the diagnosis and management of chronic coronary syndromes. Patients with coronary heart disease who have undergone CAG and OCT examinations. Exclusion criteria: ① Poor quality of OCT images; ② Underwent artificial valve replacement; ③ Severe heart dysfunction (EF < 40%), rheumatic heart disease, infective endocarditis, various congenital heart diseases, cardiomyopathy, severe arrhythmias; ④ Severe aortic stenosis, pulmonary embolism, severe renal failure (serum creatinine>250 μmol/L); ⑤ Severe electrolyte disturbance, coagulation dysfunction, and bleeding tendency; ⑥ Autoimmune diseases, malignant tumors.

### 2.3 Data collection

Collect and record general clinical information, imaging examination results, and laboratory data of the subjects. In addition, data related to the subjects’ surgeries were recorded and evaluated.

### 2.4 Screening and analysis of AVC

#### 2.4.1 Image collection

All patients underwent transthoracic echocardiography in a resting state using the UST-52105 ultrasound probe with frequencies ranging from 1–5 MHz. Imaging was performed from various windows (such as left lateral decubitus position and parasternal views). The aortic valve structure, flow conditions, and locations and extent of calcification were observed. AVC was defined as leaflet thicknes≥1 mm with any area showing high echogenicity, with or without reduced valve mobility (20).

#### 2.4.2 Grading of aortic valve calcification degree

In this study, a simpler method was used to classify AVC, based on the grading methods of AVC proposed by Bahler RC and Liu PY et al. (21, 22) (Supplementary Figure S1). The specific classification method is as follows: Level 1 AVC is defined as: a single lobe with obvious strong reflexes (calcification), while the remaining lobes exhibit Grade 2 or below; Level 2 AVC is defined as: both lobes have distinct strong reflex zones, while the remaining lobes exhibit Grade 2 or below; Level 3 AVC is defined as moderate or significant reflection enhancement in all leaflets. To evaluate inter-observer agreement, we additionally performed an agreement analysis using weighted kappa statistics, as AVC grade is an ordinal categorical variable. The results demonstrated good inter-observer agreement (weighted *κ* = 0.89). To further assess intra-observer reproducibility, a randomly selected subset of participants was re-evaluated by the same observer after an interval of at least 2 weeks. This analysis also showed good reproducibility (weighted κ = 0.90). With respect to validation against established methods, echocardiography was the primary imaging modality in the present study, and cardiac CT was not available for all participants. Therefore, a systematic comparison with CT-derived Agatston scores could not be performed in the full cohort. However, in the subset of participants with available cardiac CT data, we examined the association between echocardiographic AVC grade and CT-derived aortic valve calcium burden. A significant positive correlation was observed between echocardiographic grade and Agatston score (correlation coefficient = 0.43, *p* = 0.037) (Supplementary Table S1).

### 2.5 Coronary angiography and OCT image collection

#### 2.5.1 The analysis of CAG

Coronary angiography (CAG) During the process, the degree of native vascular stenosis was evaluated, and CAG images were independently assessed by two experienced cardiologists blinded to clinical data. Inter-observer agreement was evaluated using Cohen’s kappa coefficient.

#### 2.5.2 OCT imaging collection

After intracoronary nitroglycerin injection, OCT images were obtained using a frequency domain OCT system (St. Jude Medical, C7, USA). During imaging, contrast agent was continuously injected via a guiding catheter, with motorized pullback at 36 mm/s. Images were captured at 180 frames per second and digitized for archiving. If the catheter could not pass through the target lesion, pre-dilation with a ≤ 2 cm balloon was performed before imaging.5.3 Calcium scoring.

#### 2.5.3 OCT analysis of calcification morphology and calcium scoring

OCT analysis was performed using LightLab Image system software, based on expert consensus and previous reports (23). Calcification was measured at 1 mm intervals per OCT frame, assessing cross-sectional features. Calcification thickness was defined as the distance from the lumen edge to the calcium deposit, and its length as the distance between the proximal and distal edges. The calcification angle was measured with a protractor. Calcification was quantified using the OCT Calcification Integral Index (24). A score of 1–2 was assigned for the following: calcification angle >180° (2 points), calcification thickness >0.5 mm (1 point), and calcification length >5 mm (1 point). Total scores ranged from 0 to 4 (Supplementary Figure S2). On this basis, subjects with CAC shown by OCT were divided into two groups according to CAC severity: the 0–2 and 3–4 group.

### 2.6 Determination of plasma CTRP9 levels

Plasma levels of CRTP9 were measured using an ELISA kit purchased from Idipobioscience, following the manufacturer’s instructions carefully.

### 2.7 Experimental animals and protocols

All animal procedures complied with the Guide for the Care and Use of Laboratory Animals published by the US National Institutes of Health and were approved by the Animal Care and Use Committee of Hebei Medical College. ApoE^−/−^ mice were purchased from GemPharmatech (Nanjing, China). Eight-week-old male ApoE^−/−^ mice were randomly assigned to receive either a Western diet (WD) or a normal diet (ND) for 24 weeks to establish an aortic valve calcification model. All animals were housed in a specific pathogen-free, temperature-controlled facility under a 12-h light/dark cycle.

### 2.8 Quantitative real time-PCR (qRT-PCR) analysis

Total RNA was extracted using the Trizol reagent method (Invitrogen) and employed for first-strand cDNA synthesis. qRT-PCR was conducted using RT^2^ SYBR Green Mastermix (PARN-026Z, QIAGEN) on a 7,500 Real-Time PCR system (Thermo Fisher Scientific, Inc). The PCR protocol consisted of an initial denaturation step at 95 °C for 10 min, followed by 40 cycles of denaturation at 95 °C for 15 s and annealing/extension at 60 °C for 1 min. Gene expression levels were determined based on the CT values, and fold-changes in expression were calculated using the 2^-ΔΔCT^ method. All samples were analyzed in triplicate.

### 2.9 Western blot analysis

Mouse aortic vascular tissues were harvested and lysed to extract total protein. Protein concentrations were determined using the BCA Protein Assay Kit (Thermo Fisher Scientific, Inc. 23,227). The total proteins were separated by gel electrophoresis and transferred onto a poly-vinylidene fluoride (PVDF) membrane. The membranes were then blocked with 5% nonfat milk for 1 h, followed by overnight incubation at 4 °C with primary antibodies. After washing, the membranes were incubated with HRP-conjugated secondary antibodies at room temperature for 1 h. Protein bands were visualized using the BioRad Imaging System. Antibodies against GAPDH (#5174) and RUNX2 (#12556) were obtained from Cell Signaling Technology (Beverly, MA), while the antibody against BMP2 (Cat. No: 66383) was purchased from Sigma and the antibody against ALP (Cat. No: ab67228) was purchased from Abcam.

### 2.10 Histological staining

For histological assessment, aortic root tissues were collected from Apoe−/− mice fed a Western diet and treated with either vehicle or gCTRP9. The tissues were fixed in paraformaldehyde, embedded in paraffin, and sectioned serially for staining. Alizarin Red S staining was performed to evaluate calcium deposition in the aortic valve and aortic root lesions, and the calcified area was quantified using image analysis software. Masson’s trichrome staining was used to assess collagen deposition and fibrotic remodeling, and the fibrotic area was expressed as the percentage of collagen-positive area. Hematoxylin and eosin (H&E) staining was performed to examine overall tissue morphology and lesion architecture, and leaflet thickness was measured at the site of maximal thickening on H&E-stained sections. Quantitative analyses of total calcified area, fibrosis area, and leaflet thickness were carried out on representative sections from each animal under identical imaging and analysis settings.

### 2.11 Statistics and analysis

SPSS 26.0 and MedCalc 18.2.1 were used for analysis. Normally distributed data were expressed as x ± s and compared using t-tests or one-way ANOVA. Non-normally distributed data were presented as M (Q1, Q3) and analyzed with the Mann–Whitney U or Kruskal-Wallis H tests. Categorical data were compared using Chi-square or Fisher’s exact tests. Spearman and Kendall’s Tau-b assessed correlations. ROC curves and AUC evaluated the predictive value of CTRP9 and AVC for coronary artery calcification severity. Complete-case analysis was applied for variables with missing data. A post-hoc power analysis was performed for the CTRP9 subgroup (*n* = 340) to evaluate the adequacy of the sample size. The analysis was conducted using PASS software based on the observed effect size, and the calculated statistical power was 0.837, indicating sufficient power (>0.80) to detect the observed associations and group differences (Supplementary Table S2). *p* < 0.05 was considered statistically significant.

## 3 Results

### 3.1 General clinical data between different groups

#### 3.1.1 Baseline characteristics of the patients

A total of 900 participants were enrolled in this study. Among them, 340 participants provided additional informed consent for blood sample collection and long-term storage, and serum samples were successfully obtained for subsequent biomarker analyses. Baseline characteristics were comparable between the overall cohort of 900 participants and the subgroup of 340 participants with available serum samples, indicating that the serum subgroup was representative of the entire study population. For intergroup comparison, the 340 eligible subjects were divided into four groups according to the presence or absence of CAC and AVC. Significant differences were observed among the groups in terms of RBC, HGB, PLT, CK-MB, β2MG, ALT, GGT, HDL-C, ApoA1, Lp(a), E/e´, gender, age, hypertension, and diabetes (*p* < 0.050). The prevalence of AVC was higher in CAC patients compared to non-CAC patients (31.82% vs. 25.40%, *p* < 0.050). Additionally, the analysis of interventional surgery characteristics revealed significant differences in the number of stents, total stent length, average stent diameter, contrast volume, and radiation dose among the groups (*p* < 0.050). Notably, the CAC with AVC group required a greater number and total length of stents compared to the other groups, with statistically significant differences (*p* < 0.050). In addition, the intergroup comparison among the 340 subjects who provided serum samples across the four groups showed that plasma CTRP9 levels were significantly lower in patients with CAC than in those without CAC. Furthermore, CTRP9 levels were further reduced in CAC patients with concomitant AVC (Table 1; Supplementary Table S3).

**Table 1:** Baseline characteristics and general clinical data of each group.

Variables	Control group (*n* = 125)	CAC group (*n* = 113)	AVC group (*n* = 38)	AVC with CAC group (*n* = 64)	*p*
Male sex (*n*, %)	108 (86.40)^a^	95 (84.10)^a^	27 (71.10)^a,b^	41 (64.10)^b^	0.001
Age (y)	54.00 (47.50,64.00)^abc^	61.00 (55.00,67.00)^bc^	67.00 (61.25,71.25)	66.00 (61.25,71.00)	<0.001
Hypertension (*n*, %)	76 (60.80)	74 (65.50)	29 (76.30)	44 (68.80)	0.322
Heart failure (*n*, %)	26 (20.80)	23 (20.40)	11 (28.90)	14 (21.90)	0.717
Hyperlipidemia (*n*, %)	51 (40.80)	35 (31.00)	15 (39.50)	31 (48.40)	0.131
Diabetes (*n*, %)	41 (32.80)	44 (38.90)	14 (36.80)	32 (50.00)	0.148
Ischemic stroke (*n*, %)	15 (12.00)	11 (9.70)	2 (5.30)	9 (14.10)	0.548
Smoking (*n*, %)	55 (44.00)	48 (42.50)	15 (39.50)	17 (26.60)	0.249
Drinking (*n*, %)	47 (37.60)	30 (26.50)	10 (26.30)	16 (25.00)	0.381
HR (bpm)	70.00 (63.00,80.00)	69.00 (62.00,75.50)	67.00 (62.75,72.25)	69.00 (63.00,76.00)	0.343
SBP (mmHg)	130.00 (120.50,141.50)	132.00 (123.00,144.50)	134.50 (116.75,143.25)	132.50 (117.25,147.00)	0.895
DBP (mmHg)	79.00 (73.00,86.00)^c^	80.00 (72.00,88.50)^c^	80.00 (69.00,87.25)	75.00 (69.25,80.75)	0.032
Body mass index (kg/m^2^)	26.11 (24.49,28.41)	26.11 (24.22,28.08)	25.11 (23.43,26.93)	25.89 (24.22,27.60)	0.111
WBC (10^9/L)	6.60 (5.60,8.03)	6.20 (5.22,7.35)	6.00 (4.99,7.59)	6.55 (5.42,7.67)	0.076
NE (10^9/L)	3.93 (2.90,4.71)	3.70 (2.96,4.30)	3.47 (2.88,4.66)	3.70 (3.08,4.50)	0.360
LY (10^9/L)	2.00 (1.56,2.40)	1.85 (1.40,2.38)	1.75 (1.35,2.11)	1.86 (1.49,2.48)	0.066
RBC (10^12/L)	4.48 (4.20,4.85)^c^	4.39 (4.13,4.66)	4.38 (4.14,4.61)	4.33 (3.84,4.63)	0.003
Hemoglobin (g/L)	137.22 ± 13.61^c^	136.70 ± 12.29^c^	133.50 ± 12.73	128.53 ± 14.85	<0.001
PLT (10^9/L)	214.72 (178.00,252.50)	200.00 (165.50,238.83)	202.00 (176.25,239.25)	198.54 (175.50,232.50)	0.196
hsCRP (mg/L)	2.80 (1.10,6.70)	2.00 (1.10,5.90)	2.40 (1.45,7.65)	2.80 (1.23,6.00)	0.423
MYO (ng/mL)	51.00 (39.50,65.00)	51.00 (43.00,66.00)	58.00 (45.75,76.75)	55.00 (41.50,68.78)	0.207
CK (U/L)	71.00 (53.00,100.00)	73.00 (51.00,102.00)	74.50 (55.25,143.25)	64.50 (49.00,89.75)	0.349
CKMB (U/L)	19.00 (14.00,24.00)^b^	19.00 (14.85,23.00)	22.50 (18.75,29.50)^ac^	18.00 (13.00,21.75)	0.001
LDH (U/L)	184.00 (163.50,209.00)	182.00 (156.00,210.50)	204.00 (167.75,284.25)^c^	171.50 (154.00,204.25)	0.032
ALT (U/L)	26.50 (18.60,40.85)^a^	22.00 (14.90,31.15)	24.85 (14.18,36.03)	22.50 (16.28,32.53)	0.034
GGT (U/L)	27.00 (19.00,38.50)	23.00 (16.00,35.00)	22.00 (15.75,33.00)	23.10 (17.25,29.75)	0.091
Urea (mmol/L)	5.00 (4.26,6.20)	5.30 (4.30,6.53)	4.85 (4.20,5.66)	5.00 (4.20,6.28)	0.685
Cr (umol/L)	75.00 (65.45,86.00)	74.00 (62.50,81.50)	71.50 (64.75,83.25)	72.50 (64.00,85.00)	0.627
UA (umol/L)	350.00 (291.00,421.50)	328.30 (272.50,386.50)	313.00 (268.50,361.75)	336.00 (267.25,375.75)	0.083
β2MG (mg/L)	1.90 (1.70,2.20)^bc^	2.00 (1.80,2.30)	2.10 (1.90,2.43)	2.16 (1.90,2.40)	0.001
Total cholesterol (mmol/L)	3.73 (3.13,4.31)	3.74 (3.09,4.37)	3.94 (3.51,4.44)	3.64 (3.14,4.46)	0.351
Triglyceride,(mmol/ L)	1.54 (1.21,2.47)	1.52 (1.05,1.95)	1.54 (1.22,2.18)	1.56 (1.21,2.10)	0.428
HDL-C (mmol/L)	0.93 ± 0.20^b^	0.93 ± 0.22	0.93 ± 0.18	0.97 ± 0.21	0.183
LDL-C (mmol/L)	2.18 (1.75,2.66)	2.22 (1.62,2.78)	2.42 (1.92,2.87)	2.11 (1.78,2.70)	0.382
APoA1 (g/L)	1.07 ± 0.18^ab^	1.12 ± 0.23	1.15 ± 0.17	1.13 ± 0.22	0.045
ApoB (g/L)	0.78 (0.64,0.93)^b^	0.80 (0.61,0.97)	0.91 (0.75,1.00)	0.78 (0.66,0.99)	0.046
Lp(a) (mg/dL)	15.42 (7.65,30.64)^b^	13.30 (5.64,29.13)^bc^	26.46 (12.53,70.69)	21.68 (10.02,39.67)	0.005
Glu (mmol/L)	5.01 (4.50,5.79)	5.32 (4.64,6.29)	5.25 (4.63,6.62)	5.30 (4.66,6.19)	0.243
Ca^2+^	2.25 ± 0.09	2.24 ± 0.11	2.27 ± 0.10	2.24 ± 0.12	0.699
P (mmol/L)	1.16 (1.05,1.30)	1.13 (1.03,1.25)	1.22 (1.07,1.31)	1.19 (1.08,1.31)	0.088
LV	48.00 (45.00,52.00)	48.00 (45.00,51.00)	48.50 (46.00,51.25)	47.00 (44.25,49.00)	0.257
EF (%)	62.40 (56.45,66.85)	62.50 (57.20,66.35)	62.10 (55.80,66.80)	63.55 (60.15,66.93)	0.511
EDV	103.00 (89.00,123.00)	102.00 (88.00,123.00)	99.50 (84.00,130.50)	95.00 (83.00,108.75)	0.075
ESV	38.00 (30.00,51.00)	38.00 (29.90,48.00)	38.50 (30.00,54.10)	33.00 (28.00,41.00)	0.09
E/e´	10.75 (9.04,13.05)^c^	10.80 (8.93,13.00)^c^	13.40 (9.85,15.67)	11.59 (9.00,14.94)	0.004
Number of stent	1.00 (1.00,2.00)	1.00 (1.00,2.00)	1.00 (1.00,2.00)	2.00 (1.00,2.00)	0.424
Total stent length (mm)	29.00 (18.00,46.00)^ac^	33.00 (22.00,51.00)	30.50 (18,45.25.00)	35.00 (21.25,50.75)	0.296
Average stent diameter (mm)	3.00 (2.72,3.36)	2.83 (2.61,3.00)	2.75 (2.50,3.46)	2.75 (2.53,3.04)	0.084
Contrast volume (mL)	180.00 (160.00,240.00)	180.00 (160.00,240.00)	165.00 (160.00,242.50)	180.00 (160.00,240.00)	0.666
Radiation dose (mGy)	15132.75 (9577.50,23480.50)	15213.00 (723.00,23120.00)^c^	16352.50 (10382.25,21680.50)	16495.00 (8345.50,25912.00)	0.409
CTRP9	72.43 (61.09,84.17)^abc^	57.61 (45.07,69.74)	69.64 (51.48,87.27)	51.03 (42.25,57.60)	<0.001
Oral Aspirin	117 (93.6%)	113 (91.15%)	35 (92.1%)	57 (89.1%)	0.662
Oral Clopidogrel	85 (68%)	85 (75.2%)	22 (57.9%)	44 (68.75%)	< 0.01
Oral Ticagrelor	45 (36%)	32 (28.3%)	10 (26.3%)	25 (39.0%)	0.067
Oral ACE/ARB	36 (28.8%)	39 (34.5%)	9 (23.7%)	22 (34.3%)	0.094
Oral β blockers	97 (77.6%)	85 (75.2%)	21 (56.1%)	46 (71.9%)	< 0.001
Oral CCB	34 (27.2%)	40 (35.4%)	13 (34.2%)	20 (31.3%)	0.186
Oral diuretics	22 (17.6%)	27 (23.8%)	10 (26.3%)	14 (21.9%)	0.362

Continuous data are presented as mean ± SD or median (IQR). Categorical data are presented as *n* (%). *p* values were determined by using Kruskal-Wallis H test, one-way analysis of variance, schi-square test as an appropriate comparison. Compared with coronary calcification without aortic valve calcification group, ^a^*p* < 0.050; Compared with aortic valve calcification without coronary calcification group, ^b^*p* < 0.050; Compared aortic valve calcification with coronary calcification group, ^c^*p* < 0.050.

#### 3.1.2 Comparison of AVC detection rates among different groups with varying degrees of calcification severity

According to the above description, aortic valve calcification was divided into three levels. The results showed that the proportion of severe coronary artery calcification in the third level valve calcification group (0.846) was higher than that in the first level valve calcification group (0.065) and the second level valve calcification group (0.167) (Supplementary Figure S3).

### 3.2 Correlation and logistic regression analysis of risk factors for aortic valve and coronary artery calcification

Spearman correlation analysis revealed a weak association between aortic valve calcification (AVC) and coronary artery calcification (CAC) (*r* = 0.071, *p* < 0.05) (Table 2). In contrast, Kendall’s Tau-b correlation analysis demonstrated a significant positive correlation between severe AVC and severe CAC (τb = 0.597, *p* < 0.001) (Supplementary Table S4). In addition, we performed stratified analyses according to AVC severity (none/mild vs. moderate–severe AVC). The results showed that the association between AVC and CAC was stronger in individuals with moderate–severe AVC compared with those with none or mild AVC (Table 3). These results indicating a stronger relationship between advanced stages of both conditions.

**Table 2:** The relationship between CAC and AVC.

CAC	AVC		Total
	No	Yes	
No	125	38	163
Yes	113	64	177
Total	238	102	340
Spearman’s rank correlation = 0.071*			

*p < 0.05, **p < 0.01, ***p < 0.001. 0 < |*r*|<0.2, Extremely weak correlation or no correlation, 0.21 < |*r*| ≤ 0.40, low correlation, 0.41 < |*r*| ≤ 0.60, significant correlation, 0.61 < |*r*| < 0.80, high correlation, |*r*|>0.80, highly significant correlation.

**Table 3:** Prevalence and severity of CAC stratified by AVC severity.

Characteristics	All participants (*N* = 340)	non AVC (*n* = 238)	Mild AVC (*n* = 89)	Severe AVC (*n* = 13)	*p*
CAC presence					
CAC positive, *n* (%)	148 (52.05)	113 (47.47)	51 (57.30)	4 (30.76)	<0.001
CAC severity					
No CAC, *n* (%)	192 (56.47)	154 (52.50)	34 (38.23)	4 (30.76)	<0.001
Mild CAC, *n* (%)	115 (34.11)	65 (27.3)	48 (53.91)	2 (15.38)	
Severe CAC, *n* (%)	33 (9.70)	19 (7.98)	7 (7.86)	7 (53.80)	

Data are presented as number (%). AVC severity was classified as non-AVC, mild AVC, and severe AVC. CAC severity was categorized as no CAC, mild CAC, and severe CAC according to the predefined grading criteria. *p* values were calculated to compare the distribution of CAC presence and CAC severity across AVC severity groups.

Logistic multivariate regression analysis for AVC risk factors identified age, lipoprotein(a) [Lp(a)], and the E/e´ ratio as independent risk factors for AVC, after adjusting for potential confounders (Table 4). Similarly, a separate logistic regression analysis of CAC risk factors, conducted on a cohort of 340 subjects who underwent CTRP9 testing. A comprehensive analysis of multiple candidate variables is provided in the Supplementary Table S4, while the main text (Table 5) presents the variables that remained statistically significant. The results demonstrated that age was an independent risk factor for CAC. Moreover, CTRP9 levels were negatively correlated with CAC, suggesting its role as a protective factor against the development of coronary artery calcification. These findings underscore the complex interplay between AVC and CAC, with specific biomarkers such as CTRP9 offering potential clinical value for early detection and risk stratification.

**Table 4:** Logistic regression analysis of influencing factors of AVC.

Variables	B	SE	Wald	*p*	OR	95%CI
Age	0.088	0.010	82.296	<0.001	1.092	(1.07,1.11)
Lp(a)	0.013	0.003	17.444	<0.001	1.013	(1.01,1.02)
E/e´	0.060	0.021	7.819	0.005	1.062	(1.02,1.11)

Multivariable logistic regression analysis was performed to identify independent factors associated with aortic valve calcification (AVC). Age, Lp(a), and E/e′ were significantly associated with the presence of AVC. Regression coefficients (B), standard errors (SE), Wald statistics, *p* values, odds ratios (OR), and 95% confidence intervals (CI) are presented.

**Table 5:** Logistic regression analysis of influencing factors of CAC.

Variables	B	SE	Wald	*p*	OR	95%CI
Age	0.024	0.012	4.267	0.039	1.025	(1.00,1.05)
LDH	−0.003	0.001	7.504	0.006	0.997	(0.995,0.999)
CTRP9	−0.039	0.007	32.217	<0.001	0.962	(0.949,0.975)

Multivariate logistic regression analysis was performed to identify independent factors associated with CAC. Variables included in the final model are shown. Age was positively associated with CAC, whereas LDH and CTRP9 were negatively associated with CAC. ORs with corresponding 95% CIs are presented.

### 3.3 Predictive and diagnostic value of plasma CTRP9 levels for coronary artery calcification

**Figure 2:**
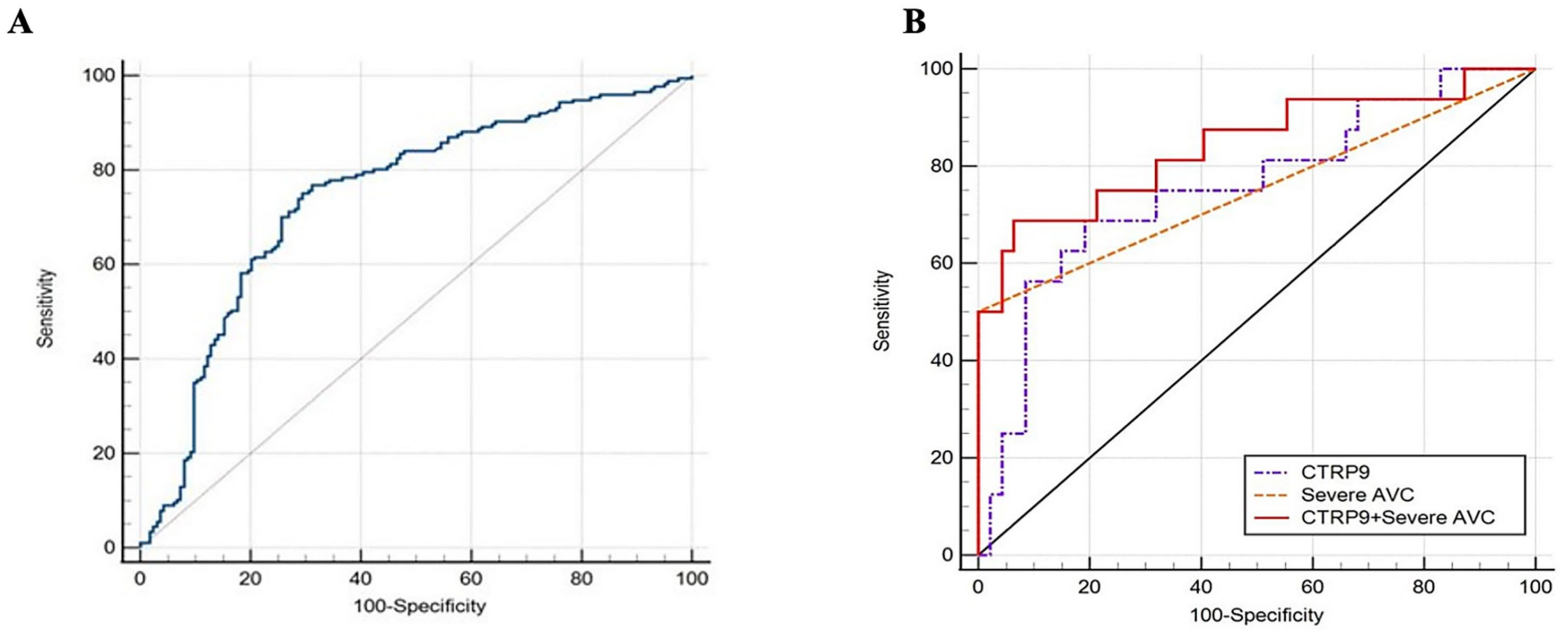
Two-panel figure showing receiver operating characteristic (ROC) analyses for prediction of severe coronary artery calcification. Panel **A** shows a single ROC curve for CTRP9, plotting sensitivity against 1 minus specificity. Panel **B** compares three ROC curves representing CTRP9 alone, severe aortic valve calcification alone, and the combined model of CTRP9 plus severe aortic valve calcification. The combined model has the largest area under the curve and the best overall discriminative performance, indicating improved predictive value when the two indicators are used together.

Receiver operating characteristic (ROC) curve analysis demonstrated that plasma CTRP9 levels possessed significant predictive value for CAC, with an AUC of 0.740 (95% confidence interval [CI]: 0.69–0.80, *p* < 0.001). At the optimal cutoff value of 64.300 ng/mL, the sensitivity and specificity were 75.1 and 70.6%, respectively (Figure 2A). In addition, stratification of subjects based on the OCT calcification scoring system (scores 0–2 vs. 3–4) revealed that plasma CTRP9 levels predicted an AUC of 0.740 (95% CI: 0.66–0.82, *p* < 0.001) for identifying higher calcification severity (scores 3–4). Notably, plasma CTRP9 levels were significantly lower in individuals with OCT scores of 3–4, suggesting that CTRP9 is negatively correlated with the severity of coronary calcification and may serve as a biomarker for risk stratification (Supplementary Figure S5). Furthermore, the diagnostic performance was enhanced when plasma CTRP9 levels were combined with the presence of severe AVC, yielding an AUC of 0.843 (95% CI: 0.715–0.972, *p* < 0.001) for predicting severe coronary artery calcification (Figure 2B).

### 3.4 Supplementation of CTRP9 markedly reduces aortic valve calcification *in vivo*

**Figure 3:**
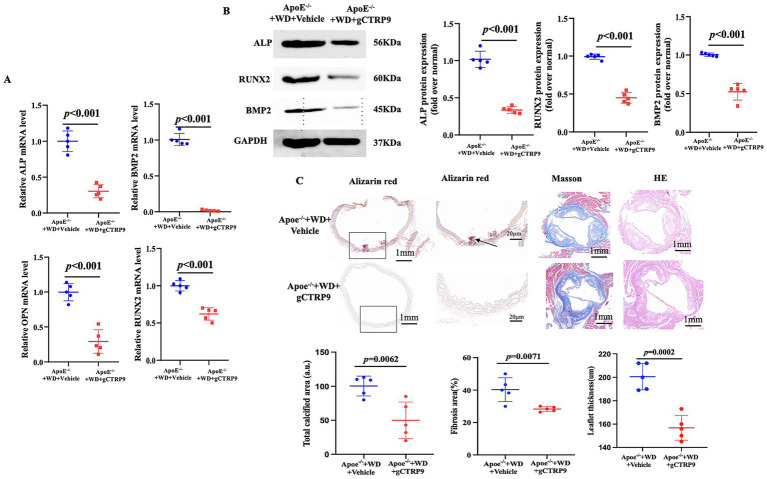
Three-panel figure showing the effects of gCTRP9 treatment on aortic valve calcification in *ApoeE*^−/−^ mice fed a Western diet. Panel **(A)** shows scatter plots of relative mRNA expression for ALP, BMP2, OPN, and RUNX2, with lower levels in the gCTRP9-treated group than in the vehicle group. Panel **(B)** shows Western blot bands and corresponding quantification for osteogenic proteins, also reduced after gCTRP9 treatment. Panel **(C)** shows histological staining images and quantification, demonstrating decreased calcification, fibrosis, and leaflet thickness in the gCTRP9-treated group compared with the vehicle group.

We have demonstrated that decreased CTRP9 levels are associated with AVC, which further correlated with increased severity of CAC severity. As the culminating step in validating the impact of CTRP9 supplementation on aortic calcification in *ApoE^−/−^* mice fed a Western diet, we administered recombinant CTRP9 (rCTRP9) via intraperitoneal osmotic pumps at a dose of 0.25 μg/g per day to these mice. Notably, the mRNA expression levels of several key calcification-related molecules, including alkaline phosphatase (*ALP*), bone morphogenetic protein 2 (*BMP2*), osteopontin (*OPN*), and runt-related transcription factor 2 (*RUNX2*), were significantly downregulated in the mice that received rCTRP9. Furthermore, compared to *ApoE^−/−^* mice maintained on the WD alone, the administration of rCTRP9 led to an improvement in the expression of calcification-related markers (Figures 3A,B). In addition to assessing the expression of calcification-related markers (e.g., Runx2 and BMP2) at the mRNA and protein levels, we also performed histological analyses of the aortic valve tissues. Specifically, H&E staining, Alizarin Red staining, and Masson staining were conducted to evaluate valve morphology, calcium deposition, and extracellular matrix remodeling, respectively. The results showed that *ApoE^−^/^−^* mice fed a Western diet exhibited marked thickening and structural disorganization of the valve leaflets, accompanied by increased calcium deposition and extracellular matrix remodeling. Importantly, treatment with gCTRP9 significantly attenuated these pathological changes, including reduced calcium deposition and collagen accumulation in the valve tissue (Figure 3C). These histological findings are consistent with the molecular results and provide direct structural evidence supporting the protective effect of gCTRP9 against aortic valve calcification.

## 4 Discussion

CAC is an active, cell-mediated process that increases with age, affecting around 50% of individuals aged 40–49 and up to 80% of those aged 60–69 (17, 25). Studies report that nearly 44% of patients undergoing PCI have CAC, with 18–24% experiencing moderate to severe forms (26). Severe CAC, particularly calcified nodules and endometrial calcifications, can stiffen coronary arteries and complicate PCI procedures, posing a challenge for interventional cardiologists (27). Moreover, CAC remains a significant risk factor for cardiovascular events, with both incidence and mortality strongly correlating with its severity (28). Despite the critical need for precise CAC assessment, current diagnostic methods have limitations. Coronary CT, while sensitive to larger calcified plaques, struggles with punctate and endometrial calcifications. Coronary angiography has low sensitivity, often missing calcified lesions. Hence, exploring new, non-invasive CAC evaluation markers is of great clinical importance.

AVC and CAC are irreversible degenerative processes that have attracted considerable attention due to their shared risk factors, including age, male gender, diabetes, hypertension, and dyslipidemia (6, 29). The early pathogenesis of both conditions involves lipid deposition and inflammation, and they commonly occur in middle-aged and elderly individuals with chronic conditions such as atherosclerosis, hypertension, and diabetes. These overlapping clinical risk factors and mechanisms suggest a potential link between AVC and CAC. Accurate measurement of CAC remains challenging. Echocardiography, however, offers a non-invasive, cost-effective, and radiation-free method with high specificity and sensitivity for AVC detection. Determining whether AVC prevalence and severity can predict CAC and its progression holds clinical importance. Owens DS et al. found AVC to be closely associated with CAC and an independent predictor of coronary events (8). However, Williams MC et al. reported a weak association between AVC and CAC scores in chest pain patients (30), while Henein M et al. found no connection between AVC and CAC in aortic stenosis patients (31). Currently, the relationship between AVC and CAC remains inconclusive, with further large-scale studies needed for validation.

In this study, 900 patients with coronary heart disease who underwent PCI and OCT examinations were included to assess the detection rates of AVC and CAC. Although common risk factors such as age and Lp(a) were confirmed for both conditions, only 31.8% of patients with CAC had concomitant AVC. Spearman rank correlation analysis showed a weak correlation between CAC and AVC (*r* = 0.071, *p* < 0.05), consistent with findings by Williams MC et al. These results suggest that AVC and CAC may involve distinct pathological mechanisms unrelated to traditional risk factors. This aligns with the findings of Razavi AC et al., who studied 325 participants without ASCVD or its risk factors and revealed that over 40% of elderly patients with significant AVC had little to no concomitant CAC (32). Additionally, Blaser MC et al. conducted a proteomic analysis of vascular calcification and AVC, identifying that AVC-enriched proteins were primarily associated with calcification, amyloidosis, apoptosis, inflammation, complement activation, and histone/nucleosome function, while proteins in carotid calcified plaques were linked to cell adhesion, migration, and cytoskeletal force generation (33). This provides valuable omics data, highlighting both similarities and differences in the progression of vascular and valve calcification.

Previous studies have shown that CTRP9 plays a key role in coronary atherosclerosis by modulating pathways involved in glucose and lipid metabolism, vasodilation, and cell differentiation. Given that CAC is a critical aspect of CAD, CTRP9 likely influences the development of CAC as well. CTRP9 has been found to inhibit aortic calcification in renal transplant recipients via AdipoR1 binding, and our earlier research indicated a reduced CTRP9 level in patients with CAC, which negatively correlates with its severity. In this study, we expanded the sample size and confirmed that CTRP9 levels were significantly lower in CAC patients. After adjusting for common risk factors, CTRP9 remained independently associated with CAC, suggesting its role as a protective factor against CAC progression in coronary heart disease patients. ROC curve analysis further demonstrated that CTRP9 has good diagnostic efficiency for CAC and may serve as a novel predictor, particularly in patients with severe calcification. Given that patients with moderate to severe calcification have a higher risk of cardiovascular events, it is crucial to accurately assess calcified lesions. We explored the correlation between plasma CTRP9 levels and severe CAC by dividing patients into two subgroups based on OCT calcification scores (0–2 and 3–4). The ROC curve for CTRP9 in severe CAC patients had an area under the curve of 0.740 (95%CI: 0.66–0.82, *p* < 0.001), with 61.8% sensitivity and 77.1% specificity. Importantly, an OCT calcium score of 3–4 indicates a severely calcified coronary lesion characterized by a large calcium arc, substantial thickness, and extended length. According to previously established OCT-based calcium scoring systems, these features are strongly associated with a high risk of stent underexpansion during percutaneous coronary intervention (PCI). Lesions with high calcium scores are often resistant to conventional balloon dilation, which may lead to suboptimal stent expansion—an important predictor of adverse clinical outcomes, including stent thrombosis and target lesion restenosis. Therefore, identifying lesions with an OCT calcium score of 3–4 has practical procedural implications. Such lesions often require dedicated plaque modification strategies (e.g., rotational atherectomy, orbital atherectomy, or intravascular lithotripsy) before stent implantation to improve lesion compliance and optimize stent expansion. Although the combination of CTRP9 and severe AVC improved the AUC compared with CTRP9 alone, this finding should be interpreted with caution. Severe AVC alone demonstrated high specificity (98.0%) but low sensitivity (21.2%) for severe CAC, indicating that it functions as a rule-in rather than a screening marker. In contrast, CTRP9 provides broader risk discrimination. Therefore, the improvement in AUC likely reflects complementary information between a circulating biomarker and imaging findings. However, because formal statistical comparison (e.g., DeLong test) and reclassification analyses (NRI/IDI) were not performed, the observed improvement should be considered preliminary. Further validation is required before clinical implementation.

Although circulating CTRP9 was measured using a commercially available ELISA kit in the present study, its current use remains more feasible in a research setting than in routine clinical practice. Commercial assays are available, but broader clinical implementation would still require assay standardization, inter-laboratory reproducibility, establishment of reference ranges, and prospective clinical validation. In our cohort, CTRP9 alone showed moderate predictive performance for severe CAC, whereas the combination of CTRP9 and severe AVC improved the discriminatory ability compared with CTRP9 alone. However, because echocardiographic AVC assessment is already widely available, non-invasive, and clinically accessible, the combined approach should currently be considered a complementary rather than a replacement strategy. At present, our findings support the potential role of CTRP9 as an adjunctive biomarker for selected-risk stratification, but not yet as a standalone test ready for routine implementation. Further external validation, assessment of incremental value beyond echocardiography alone, and cost-effectiveness analyses are needed before this combined strategy can be recommended for widespread clinical use.

In this study, we observed an interesting phenomenon: while the proportion of severe CAC was low among patients with grade 1 and 2 AVC, it reached 84.6% in those with grade 3 AVC. This suggests that more severe AVC may be linked to more severe CAC. Kendall’s Tau-b analysis confirmed a significant correlation between severe AVC and severe CAC (τb = 0.597, *p* < 0.001). Lee SE et al. similarly found that AVC progression is associated with coronary atherosclerotic plaque burden, aligning with our findings (6). Our findings showed a weak correlation between AVC and CAC when assessed as binary variables, but a much stronger concordance between severe AVC and severe CAC when disease severity was considered. This apparent discrepancy may reflect the fundamental difference between the presence of calcification and the severity of calcification, which represent different stages of disease progression. In early or mild stages, the occurrence of calcification in the aortic valve and coronary arteries may be influenced by distinct local factors and heterogeneous biological processes. As a result, the simple presence of calcification in both sites does not necessarily imply a strong overall association. In contrast, as calcification progresses, both AVC and CAC may increasingly share common pathophysiological mechanisms, including chronic inflammation, osteogenic differentiation, and systemic metabolic disturbances. Therefore, the stronger concordance observed between severe AVC and severe CAC may reflect the convergence of these shared mechanisms during advanced stages of calcific disease. These findings suggest that the presence and severity of calcification should be interpreted separately when evaluating the relationship between AVC and CAC.

In the present study, we found that gCTRP9 ameliorated vascular calcification in animals’ models. Based on our findings and previous evidence, several potential mechanisms may underlie the effects of CTRP9 on both valvular and vascular calcification. First, both AVC and CAC are characterized by osteogenic trans-differentiation of resident cells—valvular interstitial cells in the aortic valve and vascular smooth muscle cells in the coronary artery. In our experimental model, CTRP9 significantly suppressed the expression of key osteogenic transcription factors and markers, including RUNX2, BMP2, and ALP, suggesting that CTRP9 may inhibit calcification by attenuating osteogenic differentiation. This effect may be mediated through activation of the adiponectin receptor 1 (AdipoR1)–AMPK signaling pathway, which has been reported to regulate cellular metabolism and inhibit osteogenic signaling. Second, endothelial dysfunction is widely recognized as an initiating event in both AVC and CAC. Previous studies have shown that CTRP9 can enhance endothelial nitric oxide synthase (eNOS) activity and reduce reactive oxygen species (ROS) production, thereby improving endothelial function and maintaining vascular homeostasis. Third, chronic inflammation is a critical driver accelerating both valvular and vascular calcification. CTRP9 has been reported to exert potent anti-inflammatory effects, partly through inhibition of pro-inflammatory signaling pathways such as NF-κB. By suppressing inflammatory activation, CTRP9 may reduce the pro-calcific microenvironment that promotes mineral deposition and tissue remodeling. Taken together, these mechanisms suggest that CTRP9 may play a protective role in both AVC and CAC through coordinated regulation of osteogenic differentiation, endothelial function, and inflammatory responses. Although the present findings, together with prior literature, suggest that CTRP9 may influence valvular and vascular calcification through multiple pathways, including regulation of osteogenic differentiation, endothelial function, and inflammatory responses, these mechanisms were not directly tested in the current study. In particular, the proposed involvement of the AdipoR1–AMPK pathway, eNOS activation, and inhibition of NF-κB-related inflammatory signaling should be regarded as biologically plausible interpretations rather than direct mechanistic conclusions. In the present work, we primarily demonstrated that gCTRP9 attenuated calcification-related phenotypes and reduced the expression of osteogenic markers *in vivo*. Future studies incorporating direct signaling pathway analysis and *in vitro* mechanistic experiments using valvular interstitial cells or vascular smooth muscle cells will be important to validate these pathways and further clarify the molecular basis of CTRP9-mediated anti-calcific effects.

Several limitations of this study should be acknowledged. First, this was a single-center, cross-sectional observational study, which limited the sample size and precluded any firm inference of causality. More importantly, the cross-sectional design does not allow us to establish the temporal relationship between circulating CTRP9 levels and CAC development. Therefore, although lower CTRP9 levels were associated with more severe calcification, we cannot determine whether CTRP9 decline precedes CAC progression or instead occurs as a consequence of the calcific process. In addition, the possibility of reverse causation should be considered, as progressive CAC or the accompanying inflammatory and metabolic alterations may themselves influence CTRP9 production or circulating levels. Second, although our animal experiments demonstrated clear phenotypic changes together with reduced expression of key osteogenic proteins, the underlying molecular mechanisms *in vivo* were not systematically and comprehensively explored. Third, although histological staining provided direct evidence of valve calcification, quantitative imaging approaches such as micro-CT were not incorporated to more precisely evaluate calcification burden. Finally, despite adjustment for multiple covariates, residual or unmeasured confounding cannot be completely excluded. Future studies should therefore focus on validating these findings in larger multicenter prospective longitudinal cohorts, with serial CTRP9 measurements and repeated imaging assessments, to determine whether CTRP9 decline precedes, accompanies, or follows CAC progression.

In this study, the combination of CTRP9 levels and severe AVC was used to assess the severity of CAC. ROC curve analysis indicated that this combination predicted CAC severity more accurately than CTRP9 alone. Therefore, CTRP9 detection and echocardiography are recommended for patients suspected of having CHD, enabling CAC evaluation based on medical history. This approach could potentially reduce costs and aid in surgical decision-making.

## Data Availability

The original contributions presented in the study are included in the article/Supplementary material, further inquiries can be directed to the corresponding authors.
