# Supplementary material for: Circulating CTRP9 and aortic valve calcification jointly predict coronary artery calcification in coronary heart disease patients
Source: Front Nutr. 2026 Apr 29;13:1783380. doi: 10.3389/fnut.2026.1783380 (PMC13168057; doi:10.3389/fnut.2026.1783380)
Supplement: Supplementary file 1 [file Supplementary_file_1.docx]

**Table S1. Relationship between echocardiographic AVC grade and CT-derived Agatston score**

| Echocardiographic AVC grade | n | \| Agatston score, median (IQR) \| \| --- \| |
| --- | --- | --- | --- |
| No AVC, n (%) | 238 | 0(0-20) |
| Mild AVC, n (%) | 89 | 80（20-250） |
| Severe AVC, n (%) | 13 | 600（250-1400） |
| Spearmaon’s P=0.43, P=0.037 |  |  |

Note: CT-derived Agatston scores are presented as median (IQR) according to echocardiographic AVC grade. Higher echocardiographic AVC grade was associated with a higher CT-derived Agatston score. Spearman’s correlation analysis demonstrated a significant positive association between the two measures (**r = 0.43, P = 0.037**).

**Table S2. The sample size was estimated using PASS software**

| **Power Analysis Results** | | | |
| --- | --- | --- | --- |
| Alpha value (Type I error) | Beta value（Type II error） | Power value（1 – Beta） | Sample size |
| 0.05 | 0.163 | 0.837 | 340.000 |

**Note:** PASS, Power Analysis and Sample Size. Sample size was calculated using PASS software with an alpha level of 0.05, a beta value of 0.163, and a power of 0.837, yielding an estimated required sample size of 340.

**Table S3. Baseline characteristics of 900 patients**

| **Variables** | **Control Group（n=376）** | **CAC Group (n=270)** | **AVC Group**  **(n=128)** | **AVC with CAC Group(n=126)** | ***P*** |
| --- | --- | --- | --- | --- | --- |
| Male（n%） | 300(79.80)^a^ | 193(71.50)^a,b^ | 94(73.40)^a,b^ | 80(63.50)^b^ | .002 |
| Age（y） | 55.00(47.00,63.00)^abc^ | 62.00(56.00,68.00)^bc^ | 66.00(58.00,71.00) | 66.00(62.00,72.50) | <0.001 |
| Hypertension（n%） | 211(56.10)^a^ | 186(68.90)^b^ | 94(73.40)^b^ | 88(69.80)^b^ | <0.001 |
| Heart failure（n%） | 69(18.40) | 51（18.90） | 33（25.80） | 29（23.00） | .243 |
| Hyperlipidemia（n%） | 150(39.90) | 91(33.70) | 46(35.90) | 52(41.30) | .326 |
| Diabetes（n%） | 86(22.90)^a^ | 86(31.90)^a,b^ | 44(34.40)^a,b^ | 48(38.10)^b^ | .002 |
| ischemic stroke（n%） | 35(9.30) | 37(13.70) | 17(13.30) | 21(16.70) | .113 |
| Smoking（n%） | 139(37.00) | 87(33.20) | 45(35.20) | 30(23.80) | .219 |
| Drinking（n%） | 100(26.60) | 53(19.60) | 30(23.40) | 26(20.60) | .075 |
| HR（bpm） | 70.00(63.00,77.75) | 70.00(62.00,77.00) | 67.50(62.00,76.00) | 69.00(62.00,77.00) | .243 |
| SBP（mmHg） | 130.00  (120.00,141.00) | 132.00  (121.75,143.00) | 133.50  (117.25,142.75) | 132.00  (119.75,147.00) | .417 |
| DBP(mmHg) | 80.00(73.25,88.00)^c^ | 80.00(72.75,86.00) | 79.00(70.00,86..75) | 77.00(71.00,84.00) | .037 |
| BMI(kg/m2) | 26.11  (23.76,28.41) | 26.00  (24.03,27.75) | 25.75  (23.44,27.45) | 25.49  (23.22,27.34) | .142 |
| WBC(10^9/L) | 6.50(5.30,7.82) | 6.20(5.04,7.60) | 6.28(5.43,7.80) | 6.00(4.95,7.35) | .073 |
| NE(10^9/L) | 3.80(2.90,4.67) | 3.56(2.76,4.56) | 3.70(2.96,4.79) | 3.50(2.87,4.40) | .138 |
| LY(10^9/L) | 1.92(1.50,2.44) | 1.86(1.50,2.40) | 1.83(1.40,2.49) | 1.90(1.43,2.50) | .397 |
| RBC(10^12/L) | 4.46(4.14,4.76)^abc^ | 4.34(3.99,4.60) | 4.30(3.98,4.57) | 4.27(3.91,4.57) | <0.001 |
| HGB(g/L) | 137.00  (127.00,145.00)^abc^ | 133.00  (123.00,143.00) | 132.00  (122.00,138.75) | 131.00  (121.00,138.25) | <0.001 |
| PLT(10^9/L) | 214.41  (180.00,261.00)^bc^ | 202.00  (169.50,240.00) | 206.22  (170.00,242.25) | 200.00  (174.75,233.30) | .006 |
| hsCRP（mg/L） | 2.80(1.20,7.18) | 2.00(1.10,5.80) | 2.50(1.40,6.58) | 1.95(1.10,5.70) | .068 |
| MYO（ng/mL） | 52.00(41.33,68.00) | 53.00(43.00,68.00) | 56.50(45.00,73.00) | 55.00(43.75,70.00) | .173 |
| CK(U/L) | 73.00(53.00,108.75) | 73.50(52.00,102.85) | 73.00(51.00,122.00) | 68.50(49.75,95.78) | .607 |
| CKMB(U/L) | 18.00(14.00,23.00) | 18.00(14.00,23.50) | 19.35(15.23,25.98)^c^ | 18.00(13.00,22.00) | .047 |
| LDH(U/L) | 179.00  (159.00,211.50) | 182.50  (156.75,213.25) | 184.00  (163.25,221.75) | 177.00  (157.00,202.75) | .249 |
| ALT(U/L) | 24.45(16.53,37.33)^abc^ | 20.90(14.60,29.53) | 19.85(14.48,28.49) | 19.80(14.00,28.40) | .001 |
| GGT(U/L) | 26.00(18.25,38.75)^abc^ | 23.00(16.00,34.00) | 21.00(15.00,30.00) | 23.00(17.00,28.00) | <0.001 |
| Urea(mmol/L) | 5.20(4.29,6.20) | 5.22(4.30,6.50) | 5.20(4.40,6.38) | 5.40(4.30,6.46) | .461 |
| Cr(umol/L) | 72.45(63.00,84.00) | 71.20(61.75,81.00) | 73.50(65.00,83.00) | 71.00(62.75,84.00) | .607 |
| UA(umol/L) | 339.50  (282.00,411.75) | 330.00  (268.75,388.00) | 319.50  (272.93,379.50) | 324.30  (266.50,375.25) | .064 |
| β2MG(mg/L) | 2.00(1.75,2.30)^abc^ | 2.10(1.88,2.40) | 2.15(1.90,2.50) | 2.21(1.90,2.51) | <0.001 |
| TC(mmol/L) | 3.76(3.20,4.45) | 3.72(3.12,4.55) | 3.84(3.20,4.47) | 3.65(3.17,4.18) | .718 |
| TG(mmol/L) | 1.54(1.12,2.17) | 1.48(1.05,1.95) | 1.44(1.04,1.90) | 1.43(1.11,1.86) | .155 |
| HDL-C(mmol/L) | 0.94(0.82,1.08)^b^ | 0.97(0.83,1.13) | 1.00(0.86,1.13) | 0.98(0.86,1.15) | .012 |
| LDL-C(mmol/L) | 2.23(1.74,2.80) | 2.19(1.63,2.80) | 2.24(1.69,2.82) | 2.09(1.68,2.48) | .498 |
| APoA1(g/L) | 1.07(0.94,1.21) | 1.11(0.95,1.28) | 1.14(1.00,1.27) | 1.12(0.94,1.28) | .024 |
| ApoB(g/L) | 0.79(0.64,0.98) | 0.78(0.61,0.95) | 0.81(0.65,0.98) | 0.75(0.63,0.91) | .269 |
| Lp(a)(mg/dL) | 14.20(7.47,29.58)^bc^ | 12.53(6.64,28.80)^bc^ | 24.30(12.90,54.47) | 22.20(9.88,43.35) | <0.001 |
| Glu(mmol/L) | 4.91(4.43,5.78) | 5.22(4.54,6.04) | 5.11(4.44,6.16) | 5.16(4.58,6.13) | .061 |
| Ca^2+^ | 2.25(2.18,2.32) | 2.25(2.17,2.31) | 2.25(2.17,2.31) | 2.23(2.15,2.30) | .260 |
| P(mmol/L) | 1.14(1.02,1.27) | 1.13(1.02,1.26) | 1.14(1.02,1.26) | 1.13(1.04,1.27) | .905 |
| LV | 47.50(45.00,51.00) | 47.00(45.00,51.00) | 48.00(45.00,51.75) | 47.00(44.00,49.00) | .249 |
| EF(%) | 62.45(57.60,66.10) | 62.30(57.68,65.80) | 62.35(57.53,67.10) | 63.20(59.95,66.70) | .332 |
| EDV | 102.00(88.00,120.00) | 100.00(85.00,117.25) | 103.00(88.00,123.00) | 97.75(82.00,115.25) | .122 |
| ESV | 37.00(30.00,49.75) | 38.00(30.00,46.00) | 38.00(30.00,51.00) | 34.50(28.00,42.25) | .070 |
| E/e´ | 10.36(8.64,12.80)^bc^ | 10.75(8.88,13.00)^b^ | 12.00(10.00,14.55) | 11.39(9.40,14.43) | <0.001 |
| Number of stent | 1.00(1.00,2.00) | 1.00(1.00,2.00) | 1.00(1.00,2.00) | 2.00(1.00,2.00) | .001 |
| Total stent length（mm） | 24.00(18.00,42.00)^ac^ | 30.50(19.00,51.00) | 29.00(18.00,48.50) | 36.00(23.00,53.25) | <0.001 |
| Average stent diameter(mm) | 2.88(2.63,3.31) | 2.75(2.63,3.00) | 2.96(2.52,3.25) | 2.75(2.50,3.00) | .029 |
| Contrast volume(ml) | 180.00  (160.00,227.50) | 180.00  (160.00,240.00) | 175.00  (160.00,240.00) | 180.00  (160.00,252.50) | .046 |
| Radiation dose(mGy) | 9374.00  (394.00,19082.00) | 1148.00  (383.75,16958.25)^c^ | 11276.00  (429.00,19962.00) | 11293.00  (575.00,21272.75) | .033 |

Note: Continuous data are presented as mean±SD or median (IQR). Categorical data are presented as n (%). P values were determined by using Kruskal-Wallis H test, one-way analysis of variance, schi-square test as an appropriate comparison. Compared with coronary calcification without aortic valve calcification group, ^a^P<0.050; Compared with aortic valve calcification without coronary calcification group, ^b^P<0.050; Compared aortic valve calcification with coronary calcification group, ^c^P<0.050.

**Table S4 The relationship between severe CAC and severe AVC**

| CAC | AVC | | Total |
| --- | --- | --- | --- |
|  | Mild calcification | Severe calcification |  |
| Mild calcification | 69 | 2 | 71 |
| Severe calcification | 10 | 11 | 21 |
| Total | 79 | 13 | 92 |
| τb=0.597*** | | | |

Note: *,P<0.05, **,P <0.01, ***, P<0.001. 0<|τb|＜0.2, Extremely weak correlation or no correlation, 0.21<|τb|≤0.40, low correlation, 0.41<|τb|≤0.60, significant correlation, 0.61<|τb|<0.80, high correlation, |τb|＞0.80, highly significant correlation.

**Table S5. Logistic regression analysis of influencing factors of CAC**

| **Variables** | **Univariate logistic regression** | | | | |  | **Multivariate logistic regression** | | | | |
| --- | --- | --- | --- | --- | --- | --- | --- | --- | --- | --- | --- |
|  | β | S.E | Z | *P* | OR (95%CI) |  | β | S.E | Z | *P* | OR (95%CI) |
| CTRP9 | -0.04 | 0.01 | -6.16 | <.001 | 0.96 (0.95 ~ 0.97) |  | -0.04 | 0.01 | -5.68 | <.001 | 0.96 (0.95 ~ 0.97) |
| Age | 0.04 | 0.01 | 4.09 | <.001 | 1.04 (1.02 ~ 1.07) |  | 0.02 | 0.01 | 2.07 | 0.039 | 1.02 (1.01 ~ 1.05) |
| RBC | -0.15 | 0.09 | -1.74 | 0.082 | 0.86 (0.72 ~ 1.02) |  |  |  |  |  |  |
| CKMB | -0.00 | 0.00 | -1.24 | 0.213 | 1.00 (0.99 ~ 1.00) |  |  |  |  |  |  |
| LDH | -0.01 | 0.00 | -2.15 | 0.031 | 0.99 (0.99 ~ 0.99) |  | -0.01 | 0.00 | -2.74 | 0.006 | 0.99 (0.99 ~ 0.99) |
| GGT | -0.00 | 0.00 | -0.41 | 0.685 | 1.00 (0.99 ~ 1.00) |  |  |  |  |  |  |
| ALT | -0.01 | 0.00 | -1.88 | 0.061 | 0.99 (0.98 ~ 1.00) |  |  |  |  |  |  |
| β2MG | 0.44 | 0.21 | 2.11 | 0.035 | 1.56 (1.03 ~ 2.35) |  |  |  |  |  |  |
| Average stent diameter | -0.28 | 0.15 | -1.88 | 0.061 | 0.76 (0.57 ~ 1.01) |  |  |  |  |  |  |
| AVC |  |  |  |  |  |  |  |  |  |  |  |
| 0 |  |  |  |  | 1.00 (Reference) |  |  |  |  |  |  |
| 1 | 0.62 | 0.24 | 2.57 | 0.010 | 1.86 (1.16 ~ 3.00) |  |  |  |  |  |  |
| OR: Odds Ratio, CI: Confidence Interval | | | | | | | | | | | |

**Note:** Univariate and multivariate logistic regression analyses were performed to identify factors associated with CAC. Variables with statistical significance in the univariate analysis or with potential clinical relevance were included in the multivariate model. ORs and 95% CIs are presented. In the regression model, AVC was entered as a binary variable (0 = absent, 1 = present), with absence of AVC used as the reference category.
