# Supplementary material for: Circulating CTRP9 and aortic valve calcification jointly predict coronary artery calcification in coronary heart disease patients
Source: Front Nutr. 2026 Apr 29;13:1783380. doi: 10.3389/fnut.2026.1783380 (PMC13168057; doi:10.3389/fnut.2026.1783380)
Supplement: Supplementary file 2 [file Supplementary_file_2.docx]

**Figure S1.** Aortic valve calcification grading method based on Echocardiography.**
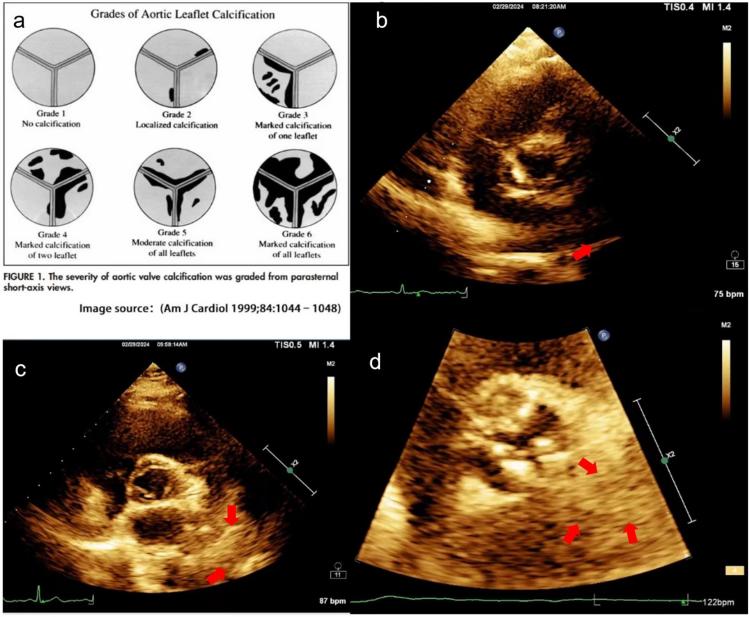
**

Note: (a) grading method for aortic valve calcification based on the number and degree of calcification in valve leaflets. (b) There is significant calcification in a single aortic valve lobe, and aortic valve calcification is classified as Level 1. (c) Two aortic valve lobes have obvious calcification, and aortic valve calcification is classified as Level 2.(d) Three aortic valve lobes have obvious calcification,and aortic valve calcification is classified as Level 3.

**Figure S2.** Calcium scoring based on optical coherence tomography.


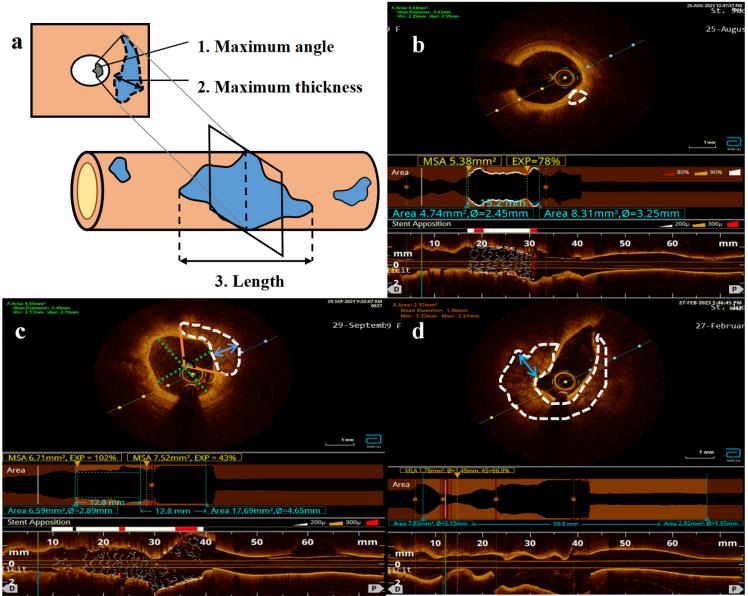


Note:(a) The scoring system model in OCT images based on the maximum angle, length, and thickness of calcification parameters. (b) Point-like calcifications with a maximum thickness and length both less than 5mm are scored as 0 points. (c) Shard-like calcifications with a maximum thickness and length both greater than 5 mm but with a maximum angle less than 180 degrees are rated as 2 points. (d) Calcifications with a maximum thickness and length exceeding 5mm, as well as a maximum angle greater than 180 degrees, are scored as 4 points.


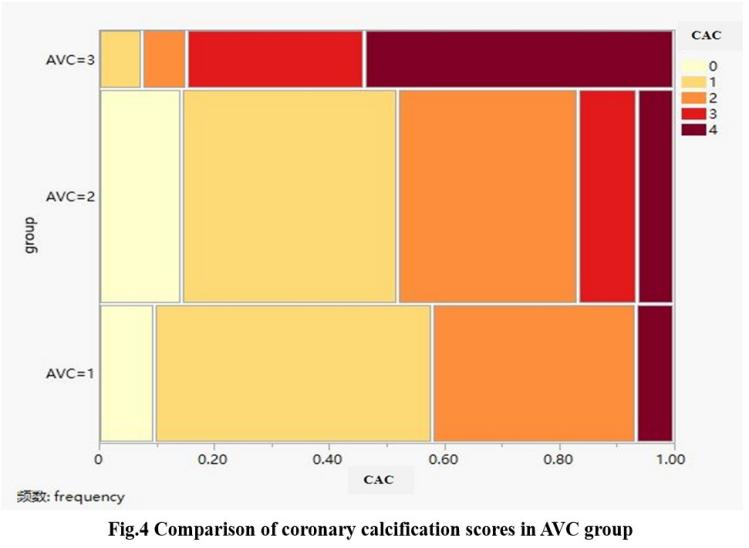
**Figure S3.** Comparison of coronary calcification scores in AVC group

**Figure S4.** The predictive value of plasma CTRP9 level for the severity of calcification.


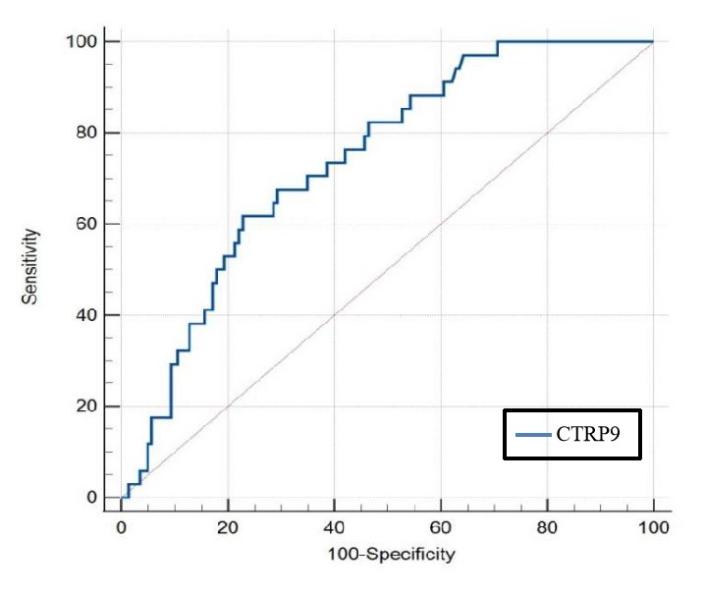
Note:The area under the receiver operating characteristic curve for Plasma CTRP9 was 0.74 (95%CI:0.66-0.82,P<0.001); the cut-off was 45.585ng/ml, the sensitivity was 61.8%, and the specificity was 77.1%.CI, confidence interval.
